# TRPV4 inhibition attenuates stretch-induced inflammatory cellular responses and lung barrier dysfunction during mechanical ventilation

**DOI:** 10.1371/journal.pone.0196055

**Published:** 2018-04-17

**Authors:** N. Pairet, S. Mang, G. Fois, M. Keck, M. Kühnbach, J. Gindele, M. Frick, P. Dietl, D. J. Lamb

**Affiliations:** 1 Immunology & Respiratory Diseases Research, Boehringer Ingelheim Pharma GmbH & Co. KG, Biberach an der Riß, Germany; 2 Department of General Physiology, University of Ulm, Ulm, Germany; 3 Institute of Immunology, Hannover Medical School, Hannover, Germany; Centre National de la Recherche Scientifique, FRANCE

## Abstract

Mechanical ventilation is an important tool for supporting critically ill patients but may also exert pathological forces on lung cells leading to Ventilator-Induced Lung Injury (VILI). We hypothesised that inhibition of the force-sensitive transient receptor potential vanilloid (TRPV4) ion channel may attenuate the negative effects of mechanical ventilation. Mechanical stretch increased intracellular Ca^2+^ influx and induced release of pro-inflammatory cytokines in lung epithelial cells that was partially blocked by about 30% with the selective TRPV4 inhibitor GSK2193874, but nearly completely blocked with the pan-calcium channel blocker ruthenium red, suggesting the involvement of more than one calcium channel in the response to mechanical stress. Mechanical stretch also induced the release of pro-inflammatory cytokines from M1 macrophages, but in contrast this was entirely dependent upon TRPV4. In a murine ventilation model, TRPV4 inhibition attenuated both pulmonary barrier permeability increase and pro-inflammatory cytokines release due to high tidal volume ventilation. Taken together, these data suggest TRPV4 inhibitors may have utility as a prophylactic pharmacological treatment to improve the negative pathological stretch-response of lung cells during ventilation and potentially support patients receiving mechanical ventilation.

## Introduction

Mechanical ventilation (MV) is an important tool for supporting patients that require assistance with breathing to improve blood oxygenation and/or carbon dioxide excretion, either as a result of medical intervention (such as surgery) or in critically ill patients. Such benefits are tempered by the potential to exert pathological forces on lung cells leading to Ventilator-Induced Lung Injury (VILI) [1]. VILI is characterized by a reduction of the alveolar epi- and endothelial barrier function resulting in pulmonary oedema formation, inflammation and alveolar flooding [2]. Two main forces act on the lung tissues and cells during mechanical ventilation, excessive volumes and/or pressures, leading to volu- or barotrauma that causes rupture of the lung parenchyma [3, 4], although end-inspiratory volume responsible for the volutrauma has been described as the main determinant of VILI rather than a barotrauma induced by an end-inspiratory pressure [5]. Lung strain during mechanical ventilation is poorly defined, especially in humans and is confounded by the heterologous local lung susceptibility during MV [6, 7]. During MV injured regions of the lung will receive smaller fractions of the total tidal volume from the inspired tidal volumes, e.g. due to alveolar collapse and fluid extravasation, therefore other lung areas will receive the majority of the tidal volume leading to massive overdistension of this areas and local damage perhaps even with protective ventilation strategies [6, 8]. In turn areas that receive the higher tidal volume, may promote a local inflammatory response that triggers a generalized inflammatory response in the lung tissue [6, 9]. A mechanism of injury, termed “biotrauma”, has been elaborated postulating that the stress produced by mechanical ventilation through overdistension of lung units not only exacerbate but also may initiate an inflammatory response [6, 10]. Loss of the alveolar-capillary barrier due to the mechanical forces results in losing the compartmentalization of the local pulmonary response and releasing pro-inflammatory mediators into the systemic circulation leading to MSOF [11, 12]. Ranieri et al. [13] support this concept by demonstrating that the concentration of pro-inflammatory cytokines in both bronchoalveolar lavage fluid (BALF) and serum could be decreased with a lung-protective ventilation strategy. How the stress induced by mechanical ventilation is converted by lung cells into the response seen in VILI is still unknown. In the lungs cytokines are produced by alveolar macrophages but also by bronchial, bronchiolar and alveolar epithelial cells [6, 14, 15]. Previous studies have demonstrated that most alveolar cells are capable of producing pro-inflammatory mediators such as tumor necrosis factor (TNF)-α, interleukin (IL) -6, IL-8 and IL-1β when stretched *in vitro* or when ventilated in *ex-vivo* experiments (nicely reviewed in [5]).

The potential involvement of cation channels in mediating the inflammatory response generated in the lung after mechanical stress has been demonstrated in isolated rat lungs in which the increase in microvascular permeability was abolished by gadolinium (inhibitor of stretch-activated nonselective cation channels) [16].

The force-sensitive transient receptor potential vanilloid ion channel (TRPV4) [17] is a Ca^2+^-permeable cation channel expressed in many tissues including pulmonary bronchiolar and alveolar epithelia, alveolar macrophages and endothelium [18, 19]. TRPV4 activation has also been reported to induce inflammatory pathways in immune cells and to induce pro-inflammatory cytokines/chemokines secretion in response to lipopolysaccharide (LPS) in epithelial cells [20, 21]. Macrophages TRPV4 has also been showed to regulate pro-inflammatory cytokine secretion [21, 22]. Furthermore TRPV4 has been implicated to play a role in ARDS. It has been demonstrated to mediate the lung injury response to a sterile stimulus *in vivo* in mice exposed to hydrochlorid acid (HCL), assessed by lung permeability increase, inflammatory cell influx and pro-inflammatory cytokine levels (e.g. IL-1ß, KC, MCP-1, RANTES, IL-6) [21, 23, 24]. Protection from acute lung injury response to HCL was observed in TRPV4 KO mice or in mice treated with different small molecule TRPV4 inhibitors [21, 23, 24]. In addition to TRPV4´s effect on the cytokine/inflammatory change in ARDS, TRPV4 activation can induce lung endothelial/epithelial barrier dysfunction [18, 25]. Additionally, TRPV4 has been linked to play a critical role in cellular transduction of mechanical forces, getting activated by cell swelling, surface expansion, stretch and in lung microvessels by hydrostatic stress (presumably by circumferential vessel stretch) [26–30].

A recent study in fetal mouse distal lung epithelial cells demonstrated that TRPV4 may also play an important role in the transduction of mechanical signals in the lung epithelium during ventilation by modulating the stretch-induced release of pro-inflammatory cytokines [31].

The present work focuses on the effect of lung cell stretch due to the overdistention during ventilation (with high tidal volumes). Because TRPV4 activation has been implicated in modulating an inflammatory response in lung epithelial cells and macrophages and has been suggested to be activated by mechanical forces, we hypothesized that a selective inhibitor of TRPV4 could prophylactically improve the negative effects of mechanical ventilation. To test this hypothesis we used the TRPV4 antagonist GSK2193874, an orally active, potent and selective blocker of TRPV4 [32, 33] and investigated the role of TRPV4 in cell strain induced inflammatory response in human epithelial cells and macrophages *in vitro* and further test its potential to improve lung permeability increase and inflammatory response during mechanical ventilation *in vivo*.

## Methods

### Ethics statement

All human blood samples were obtained from volunteers with prior written informed consent. The blood donation was voluntary and in compliance with data protection standards. Please note that the current process of the donation service at Boehringer Ingelheim in Biberach is approved by the appropriate external review board of the federal state of Baden-Württemberg, Germany named "Ethik-Kommission der Landesärztekammer Baden-Württemberg". Blood donation from healthy subjects is voluntary, ensure data protection for the participants and prior written informed consent is obtained from each blood donor. The same principles applied to the blood donation at the time of blood sampling for the current manuscript. Please note that we have obtained blood from less than 5 healthy volunteers for the purpose of our investigation and the blood draw was not part of a clinical trial but for basic research purposes only. Therefore, no prospective ethics approval was necessary for the blood donation. In any case, the principles of voluntary donation, data protection and prior written informed consent were applied.

Human cells sourced from commercial vendors were verified to have associated signed informed consents in place. This process was endorsed by a panel of senior company scientists and physicians."

### Calcium 6 assay on the FLIPR^TETRA^

Pharmacological activation and inhibition of TRPV4 was analysed using the FLIPR Calcium 6 Assay kit (molecular devices #R8191 bulk kit) and was performed according to the manufacturer’s instructions. Briefly lung epithelial cells NCI-H292 (Cat. No. CRL-1848^TM^ from the American Type Culture Collection ATCC, Manassas, VA) were seeded with a density of 3 x 10^4^ cells/well in 25 μL medium/well (RPMI-1640 medium, Gibco, Grand Island, N.Y., containing 10% heat-inactivated fetal bovine serum) on assay plates (384 well Poly-D-Lysin black/clear bottom, Biocoat #4663) and incubated for 24 h. Cells were washed 3 times with assay buffer (HBSS [+ CaCl_2_/MgCl_2_] + 20 mM Hepes + 0.1% BSA; pH 7,4) and incubated for 2 h with the calcium 6 dye solution (2.5 mM Probenecid and calcium 6 dye in assay buffer according to the manufacturer’s instruction) at 37° C in 5% CO_2_, humidified air. For fluorescence measurement cells were transferred to the FLIPR and the TRPV4 antagonist GSK2193874 was given with 10 μl/well with different concentration and cells were preincubated for 15 min during read out (FLIPR^TETRA^, Molecular Devices, excitation 470–495 nm, emission 515–575 nm, with 2 read intervals, first read interval with 1 read per second for 10 s before antagonist dispersion and 1 read per second after antagonist addition for 50 s and a second read interval with 1 read every 10 s for 84 times) before agonist addition. Afterwards cells were stimulated with 10 μl/well of the TRPV4 agonist GSK1016790A (2 nM) during read out (FLIPR^TETRA^, Molecular Devices, excitation 470–495 nm, emission 515–575 nm, with 2 read intervals, first read interval with 1 read per second for 10 s with 1 read before and 9 reads after agonist dispersion and a second read interval with 1 read every 3 s for 210 times) and the concentration-dependent inhibition of calcium influx was determined.

### TRPV4 agonism effect on cytokine release

NCI-H292 cells were seeded with a density of 5 x 10^4^ cells/well with 200 μL RPMI-1640 medium (Gibco, Grand Island, N.Y.) containing 10% heat-inactivated fetal bovine serum (FBS) on 96 well culture plate (Nunclon^TM^ Delta Surface, Thermo scientific) and incubated at 37° C in 5% CO_2_, humidified air for 24 h. Afterwards medium was removed and cells were preincubated for 1 h in the presence or absence of the TRPV4-Antagonist GSK2193874 [1μM] in 200 μL medium. Medium was removed one more time and cells were incubated at 37° C in 5% CO _2_, humidified air for 24 h in 200 μL medium in presence or absence of a non-cytotoxic dose of the TRPV4 agonist GSK1016790A [3nM]. Than supernatant was collected and stored at −80°C for later analyses.

### Uniaxial cell strain and microscopy

Uniaxial cell strain was performed on the Stretch/compression device (University Ulm, Ulm, Germany), a device for simultaneous live cell imaging during uni-axial mechanical strain or compression [34]. An elastic silicon membrane (Specialty Manufacturing, Saginaw, MI 48603–3440 USA) was cut into a rectangular piece (9x2cm) and clamped into the membrane holders that shape the membrane into a chamber (see Stretch/compression device) [34], autoclaved and coated overnight at 4°C with fibronectin (5μg/ml in PBS, both from Sigma-Aldrich, Steinheim, Germany). Human lung epithelial cells (NCI-H292) were seeded in the elastic silicon chamber (4x10^5^/membrane) and cultivated in medium at 37° C in 5% CO_2_, humidified air for 24h. Prior to imaging, the cells were pre-incubated in medium at 37°C/5% CO_2_ with 2μM of the fluorescent Ca^2+^ dye fluo-4 and 0.2% Pluronic F127 (Molecular Probes; Karlsruhe, Germany), protected from light with or without the TRPV4 antagonist GSK2193874 (1μM) for 30 min and another 30 min at RT. For cell stretch, the medium was replaced with bath solution (pH 7.4; 140 mM NaCl, 5 mM KCl, 1 mM MgCl_2_, 2 mM CaCl_2_, 5 mM glucose, and 10 mM HEPES; all from Sigma-Aldrich). Then the membranes were fastened onto the stretch apparatus [34], mounted on a Zeiss Axiovert 200 (Carl Zeiss, Oberkochen, Germany) with a 20X plan Neofluar Zeiss objective. Images were acquired with a CoolSnap EZ CCD camera and Metamorph software (exposure time of 30ms and an acquisition rate of 0.5 frames per second) and an EGFPfilter cube (excitation 470/20 nm, emission 525/25 nm, dichroic 490 nm). The membranes were stretched at RT with a triangular waveform one single time from 0% to 80% length increase and back to 0% within 800 ms.

The average grey values in the image sequence were determined with ImageJ [35] by drawing a region of interest that comprised the adherence area of a single cell. To compensate for the slight sideward shift of the cell after the strain, the region of interest was manually repositioned. Data were transferred to MS-excel and after background subtraction, the average fluorescence values of each cell before and 10 s after the strain were determined. The strain-induced change after stimulation was expressed as the % change intensity compared to baseline signal before stretch.

### Equibiaxial cell strain

The Flexcell FX-5000 Tension System (FX5K®; Flexcell International Corp, Hillsborough, NC) was used to apply mechanical cyclic tensile stretch on lung epithelial cells (NCI-H292) and macrophages. The FX5K® is a computer-based system that uses a vacuum to strain cells adhered to flexible silicon membranes (BioFlex® plates; Flexcell International Corp) arranged in a format of six wells per plate with a total growth area of 9.62 cm^2^ per well. The deformation of the flexible membrane also causes the attached cells to deform. NCI-H292 cells were seeded onto the Collagen Type I-coated BioFlex® plates at a density of 1 × 10^6^ cells/well, macrophages at a density of 2 x 10^6^ cells/well. Cells were exposed to continuous mechanical stimulation with an equibiaxial half sinusoidal waveform with an elongation from 8% to 30% and a frequency of 1.25 Hz for up to 48 h at 37° C in 5% CO_2_, humidified air. Control cultures were grown under the same conditions but without the strain protocol. Than supernatant was collected and stored at −80°C for later analyses.

### Human monocyte derived macrophages

Human whole blood was obtained from anonymised healthy volunteers. Blood was donated by internal donors at the centre for occupational health at Boehringer Ingelheim in Biberach. The donors provided signed informed consent that allows use for scientific purposes. Peripheral blood mononuclear cells (PBMCs) were isolated by means of density gradient centrifugation using Ficoll-Paque™ and a Leucosep Tube (Greiner Bio-One GmbH) according to manufacturer’s instructions. CD14 positive monocyte purification was performed by magnetic activated cell sorting (MACS) according to the manufacturer’s instructions (Monocyte Isolation Kit II, Miltenyi Biotec) and seeded 2x10^6^ cells/cm^2^ in XVIVO-10 medium (Lonza) on Collagen Type I-coated BioFlex® plates. Medium was supplemented with either 100 ng/mL Granulocyte-Macrophage Colony Stimulating Factor (GM-CSF) to induce an M_1_ phenotype or Macrophage Colony Stimulating Factor (M-CSF) to induce an M_2_ phenotype for 7 days. Differentiated macrophages on BioFlex® plates were than preincubated in presence or absence of the TRPV4 antagonist GSK2193874 [1μM] at 37° C in 5% CO_2_, humidified air for 1 h and introduced in the Flexcell FX-5000 Tension System for cell strain. Macrophages were exposed to continuous mechanical stimulation with an equibiaxial half sinusoidal waveform with an elongation from 8% to 30% and a frequency of 1.25 Hz for up to 48 h at 37° C in 5% CO_2_, humidified air. Control cultures were grown under the same conditions but without the strain protocol. Than supernatant was collected and stored at −80°C for later analyses.

### Murine mechanical ventilation model

All experimental procedures were performed in accordance with European and local animal welfare regulations and approved by the Regierungspräsidium Tübingen in Germany with the approved animal experimental license TVV 13-014-02.

Experiments were performed in female Balb/c mice (n = 82; Charles River, Sulzfeld, Germany) aged 10–12weeks, with weights ranging from 23 to 28 g. The TRPV4-Antagonist GSK2193874 [90mg/kg] or the solvent (0.5% Natrosol with 0.015% Tween80 in Covaris S2) were administered orally by gavage 1 h before ventilation. Mice received intraperitoneal narcoren (60 mg/kg) and rompun (2.5 mg/kg) with additional dosing as needed to maintain appropriate anesthesia. A cannula (Fa. Harvard, USA, Art.-Nr.: NP73-2836) was inserted into the trachea, sutured, and coupled to a flexiVent (SCIREQ, Montreal, Quebec, Canada) small animal ventilator and controlled with the Software FlexiWare (Fa. EMKA Technologies, Paris, France). Mechanical ventilation was performed with different ventilation protocols; tidal volumes of 20 ml/kg (control n = 5; treated n = 4) and 30ml/kg (control and treated n = 8) with a frequency of 75/min and 2cm H_2_O PEEP and a control group ventilated with a normal tidal volume of 6.5ml/kg (n = 4) and a frequency of 150/min and 3 cm H_2_O PEEP for 3h and an additional unventilated control group (n = 6). During the experiments mice were placed on thermostatically-controlled heat mats to preserve body temperature. After ventilation mice were euthanized with an overdose of anesthetic (ventilated until cardiac arrest). Bronchoalveolar lavage (BAL) was performed using 2 times 0.8 ml Hanks Salt Solution (Fa. Biochrom AG) and 0.6 mM EDTA (Fa.: Promega). The bronchoalveolar lavage fluid (BALF) was centrifuged at 1500 rpm at 4°C for 10 minutes and the supernatant was stored at −80°C for later analyses.

### Pierce™ BCA Protein Assay Kit

For total protein concentration measurement in BALF supernatant the Thermo Scientific Pierce BCA Protein Assay Kit was used. Assays were performed according to the manufacturer’s instructions.

### ELISA/MSD

For cytokine measurement in cell culture supernatants and BALF supernatant the Mesoscale Discovery V- and U-PLEX multiplexing technology was used. V-PLEX plates were used in the Chemokine and Pro-Inflammatory configuration. U-PLEX plates were individually spotted with the antibody pairs against the desired analytes. Assays were performed according to the manufacturer’s instructions.

### Calculations & statistics

For statistical analyses Graph Pad Prism Software for Windows version 7 was used. Significance levels are shown as * p ≤ 0.05; ** p < 0.01; *** p < 0.001; **** p < 0.0001 or “ns” for not significant (p > 0.05); ANOVAs were corrected for multiple comparisons with a Tuckey correction.

## Results

### Effect of TRPV4 agonism on cells Ca^2+^ influx

**Fig 1:**
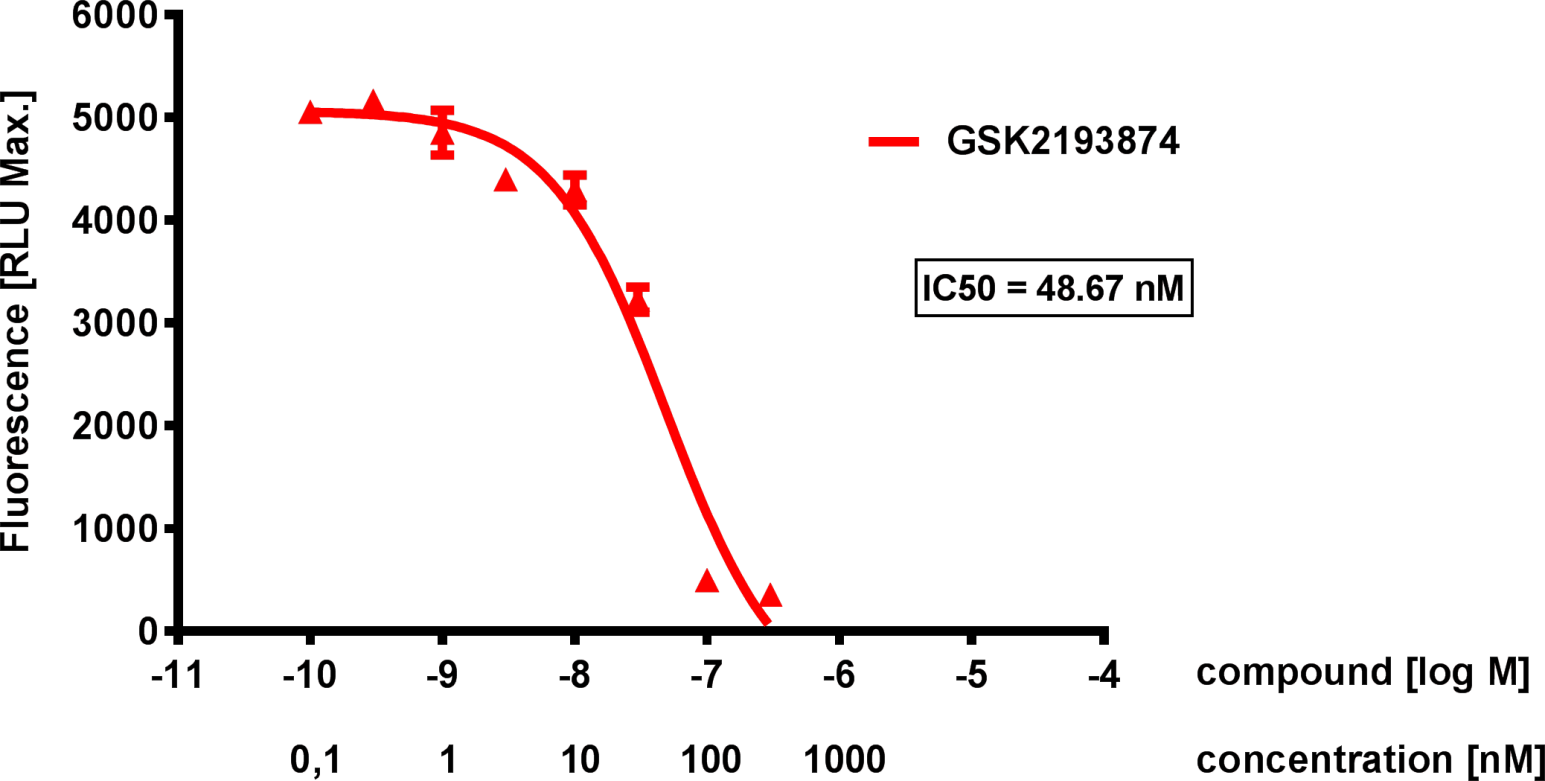
Concentration-dependent inhibition of TRPV4 effect on Ca^2+^ response. Representative Ca^2+^-Influx measurement in NCI-H292 cells stimulated with the TRPV4 agonist GSK1016790A (2 nM) and challenged against different concentration of the TRPV4 antagonist GSK2193874 (0.1 nM, 0.3 nM, 1 nM, 3 nM, 10 nM, 30 nM, 100 nM and 300 nM) preincubated for 15 min before agonist addition in the FLIPR^TETRA^. Concentration-dependent inhibition of the agonist effect through TRPV4 antagonism with an IC50 of 48,67 nM. Data are mean ± SEM; n = 3.

To investigate the effect of TRPV4 on intracellular calcium responses, human lung epithelial cells (NCI-H292) were incubated with the TRPV4 agonist GSK1016790A (2 nM) and intracellular calcium influx was measured. A significant increase in intracellular calcium concentration was observed after TRPV4 agonist addition (with EC50´s ranging from 1–2 nM, data not shown) and the significant increase in calcium after agonist addition (2 nM) was concentration-dependently inhibited by the TRPV4 antagonist GSK2193874 with an IC_50_ of approximately 50 nM and an IC_95_ of approximately 1 μM (Fig 1). In subsequent studies, 1μM was used as a maximal efficacious concentration, but not a supra-physiological concentration.

### Effect of stretch on cells Ca^2+^ influx

**Fig 2:**
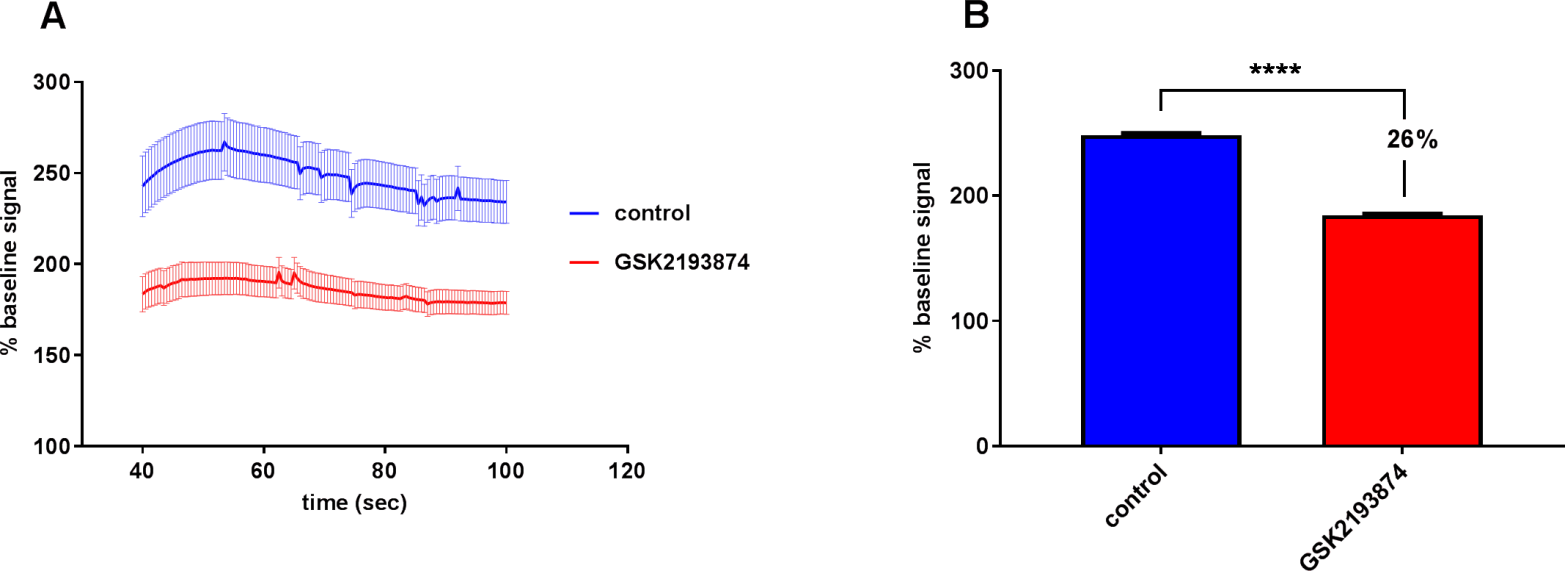
TRPV4 mediated calcium influx after stretch. Ca2+ response in NCI-H292 cells 10 s after a single uni-axial cell-stretch to 80% length increase and back to relaxation within 800 ms. Cells were loaded with the Ca^2+^ dye fluo-4 (2**μ**M) and 0.2% Pluronic F127 (Molecular Probes; Karlsruhe, Germany) and the average fluorescence values of each cell before and 10 s after the strain were determined. The strain-induced change after stimulation was expressed as the % change intensity compared to baseline signal before stretch. (A) Ca^2+^ response 10 s after stretch for 60 seconds. (B) Summary of the mean % Ca^2+^ response from the 60 sec after stretch, a 2.5 fold increase in the [Ca2+]_i_ was observed that was significantly decreased by 26% with the TRPV4 antagonist (1 **μ**M) GSK2193874 (184.5 ± 5.07 vs 248.6 ± 10.17). For (A) and (B) data are mean ± SEM; (control n = 121; GSK2193874 n = 94, summary of 13 experiments; ****p ˂ 0.0001 vs control; Unpaired two-tailed t test).

To investigate whether TRPV4 is involved in the mechanical strain induced stress response in NCI-H292, we studied the Ca^2+^ response after uni-axial cell-stretch with various combinations of stretch speeds and distances. After a single stretch to 80% length increase and back to relaxation within 800 ms a 2.5 fold increase in the intracellular Ca^2+^ concentration was observed that was significantly decreased by 26% with the TRPV4 antagonist GSK2193874 (184.5 ± 5.07 vs 248.6 ± 10.17) (Fig 2A and 2B).

### Effect of TRPV4-agonist on cell cytokine release

**Fig 3:**
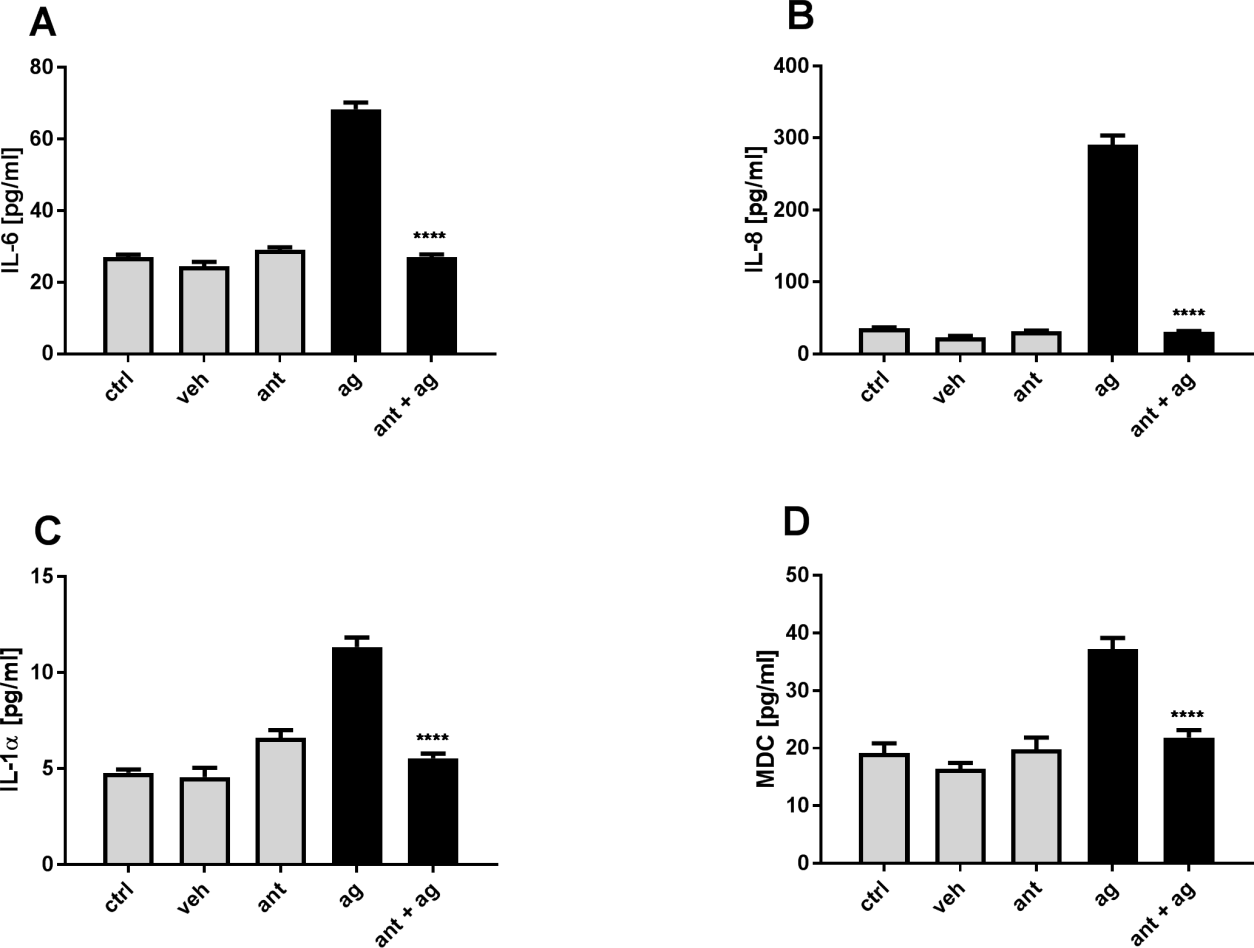
TRPV4 mediated cytokine release. Representative experiment of NCI-H292 cells incubated for 24 h in the presence or absence of the TRPV4 agonist GSK1016790A [3nM] (ag) with or without pre-treatment with the TRPV4-Antagonist GSK2193874 [1**μ**M] (ant). (A,B) release of IL-6 and IL-8 through TRPV4 activation compared to medium (ctrl) and DMSO control (veh) that could be blocked by the TRPV4 antagonist. (C,D) TRPV4 mediated release of IL-1α and MDC. Data are mean ± SEM; (n = 6; ****p ˂ 0.0001 vs agonist control; one-way ANOVA Tukey's multiple comparisons test).

To investigate the functional consequences of an increase in intracellular calcium concentration, the effect of the TRPV4 agonist GSK1016790A on NCI-H292 human lung epithelial cells was examined for cytokine release. NCI-H292 cells were incubated for 24 h in the presence or absence of a non-cytotoxic dose of GSK1016790A [3nM] with or without treatment with the TRPV4-Antagonist GSK2193874 [1μM]. GSK1016790A [3nM] increased release of IL-6 by 2.8 fold and IL-8 by 12.4 fold and this effect could be completely blocked by the TRPV4 antagonist (Fig 3A and 3B). The TRPV4 agonist also induced a 2 fold increase in IL-1α and MDC that was abolished by TRPV4 antagonism (Fig 3C and 3D). Other cytokines were measured without significant effect (IL-12p70, IL-17A, IL-18, IL-1ß, MCP-1, TNF-α).

### Effect of TRPV4 antagonism on stretch induced cytokine release

**Fig 4:**
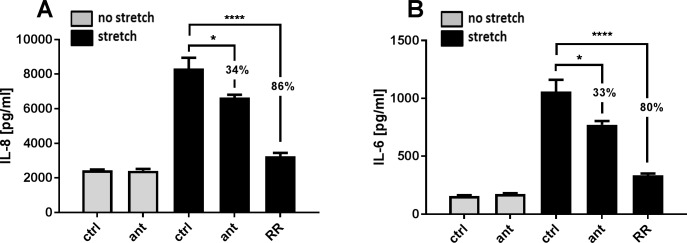
TRPV4 mediated stretch-induced cytokine release. Repesentative experiment of NCI-H292 cells seeded on silicoelastic membranes and exposed to cyclic equibiaxial stretch (cyclic 30% strain with 1.25 hz) for 24 h in the presence or absence of the TRPV4-Antagonist GSK2193874 [1**μ**M]. (A) Stretch induced release of IL-8 compared to unstretched control (ctrl) reduced by 34% with the TRPV4 antagonist (ant) and reduced by 86% with Ruthenium Red (RR). (B) IL-6 release via stretch that was reduced through TRPV4 antagonism by 33% (ant) and reduced by 80% with Ruthenium Red addition (RR). Data are mean ± SEM; (n = 3; *p ˂ 0.05; ****p ˂ 0.0001 vs stretch control; one-way ANOVA Tukey's multiple comparisons test).

We further investigated the effect of mechanical stretch on cytokine release and the role of TRPV4 in the airway epithelial response. NCI-H292 cells seeded on a collagen I coated silicoelastic membrane were exposed to a cyclic stretch with an amplitude of 20% and a frequency up to 0.5 Hz for 24h. Both IL-6 and IL-8 release were marginally increased compared with unstretched cells (data not shown). Increasing the stress stimulus with a cyclic strain of 30% (from a minimum strain of 8% to a maximum of 30%) and a frequency of 1.25 Hz for 24 h, resulted in an 3.4 fold increase of IL-8 release and an 6.8 fold increase of IL-6 release compared to unstretched cells (Fig 4A and 4B). The stretch induced IL-8 increase could be reduced by 34% with the TRPV4 antagonist [2 μM] and was decreased by 86% by the general TRP-channel blocker Ruthenium Red [10 μM] (Fig 4A). A similar observation was seen with IL-6 (Fig 4B). These data showed that a high magnitude mechanical stretch results in a cytokines IL-6 and IL-8 release in NCI-H292 epithelial cells and that this effect is significantly reduced by 30% by TRPV4 antagonism but also suggest that other mechano-sensing channels could play a role in the secretion of cytokines induced by mechanical strain. Other cytokines were measured without significant effect (IL-18, IL-17A, IL-1α, IL-1ß, MCP-1, TNF-α).

### Effect of stretch on macrophages cytokine release

**Fig 5:**
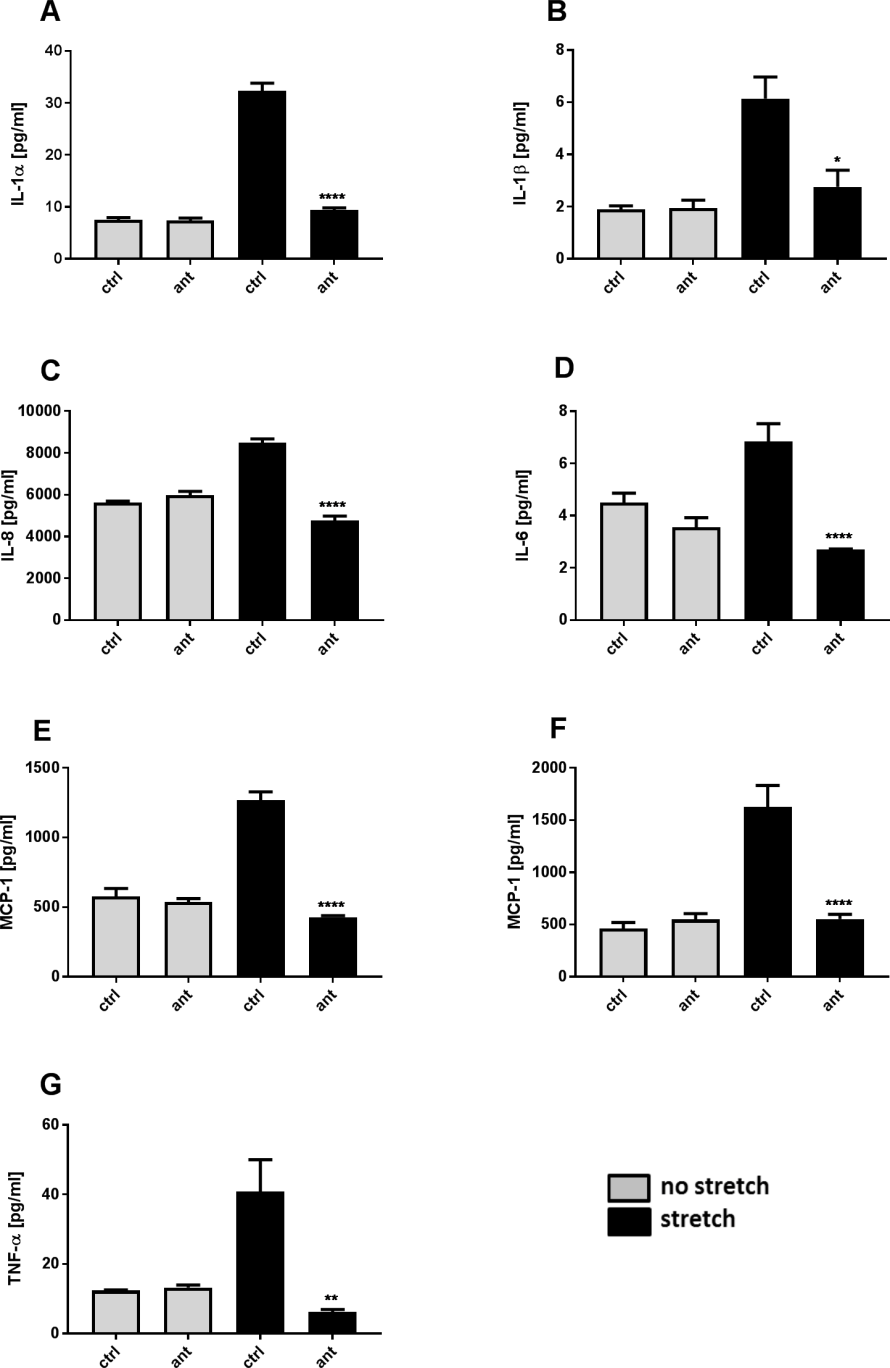
TRPV4 mediated stretch-induced cytokine release in macrophages M1 and M2. Representative experiment of Macrophages seeded on silicoelastic membranes and exposed to cyclic equibiaxial stretch (cyclic 30% strain with 1.25 Hz) for up to 48 h in the presence or absence of the TRPV4-Antagonist GSK2193874 [1**μ**M]. (A-E) Stretch induced cytokine release in M1 macrophages compared to unstretched control (ctrl) that could be blocked with the TRPV4 antagonist (ant). Stretch induced release of MCP-1 and TNF-α in M2 macrophages that could be blocked by TRPV4 inhibition (F,G). Data are mean ± SEM; (n = 3; *p ˂ 0.05; **p ˂ 0.01; ***p ˂ 0.001; ****p ˂ 0.0001 vs stretch control; one-way ANOVA Tukey's multiple comparisons test).

Human monocytes were seeded on silicoelastic membrane and differentiated with G-MCSF (M1) or M-CSF (M2) for seven days. Macrophages were exposed to the same equibiaxial stretch protocol (cyclic 30% strain with 1.25 hz) as for the lung epithelial cells, but significant cytokine release was observed after 36 h and 48 h. The mechanical stretch induced stress on the macrophages (M1) resulted in increases in IL-1α (4.3-fold; 48h; Fig 5A), IL-1β (3.2-fold; 48h; Fig 5B), IL-6 (1.5-fold; 48h; Fig 5C) IL-8 (1.5-fold; 48h; Fig 5D) and MCP-1 (2.2-fold; 36h, Fig 5E) compared to unstretched cells. All cytokine release was abolished, by the TRPV4 antagonist [2 μM]. M2 macrophages showed an about 3 fold increase in MCP-1 after 36 h and an about 2 fold increase of TNF-α after 48h stretch that were both blocked by TRPV4 antagonism (Fig 5F and 5G) but no significant increases in IL-1a, IL-1b, IL-6 or IL-8. Other cytokines were measured without significant effect (IL-10, IL-12p70, IL-2, MDC).

### TRPV4 antagonist effect on ventilation induced cytokine release and protein concentration in BALF

**Fig 6:**
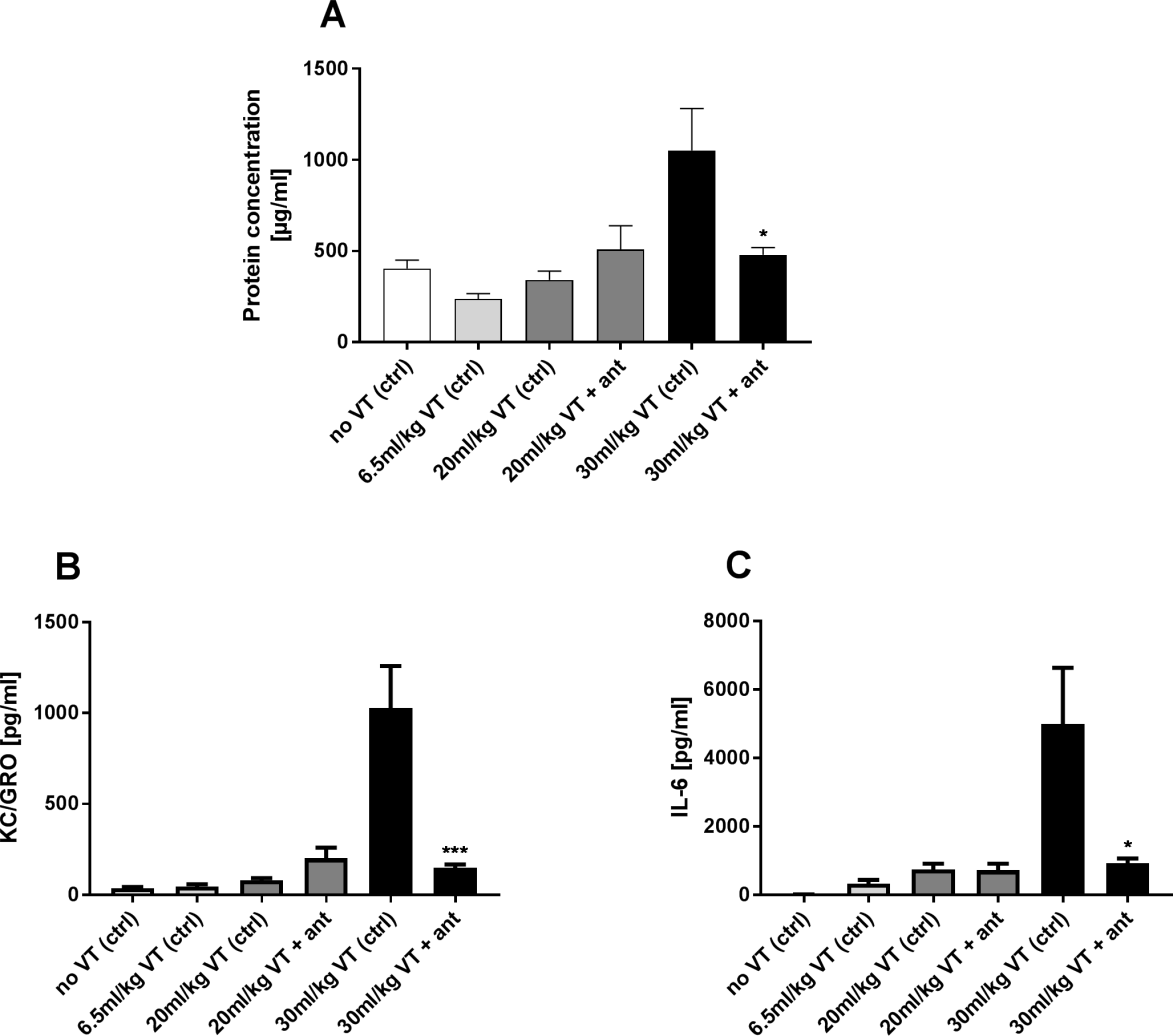
TRPV4 antagonist effect on ventilation induced cytokine release and protein concentration in BALF. Balb/c mice were anesthetized and mechanically ventilated (VT) in presence or absence of the TRPV4 antagonist GSK2193874 [90mg/kg] with different ventilation protocols; with tidal volumes of 20 ml/kg and 30ml/kg with a frequency of 75/min and a control group (ctrl) ventilated with a normal tidal volume of 6.5ml/kg and a frequency of 150/min for 3h and a non-ventilated control group (ctrl). (A) Increased protein concentration in BALF was observed at 30 ml/kg ventilation that could be blocked by TRPV4 inhibition (ant). (B,C) Increase release of the cytokine KC/GRO and IL-6 after a 30 ml/kg ventilation that could be blocked by TRPV4 antagonism (ant). Data are mean ± SEM; (n = 8); *p ˂ 0.05; ***p ˂ 0.001 vs 30 ml/kg VT control; one-way ANOVA Tukey's multiple comparisons test).

After showing that TRPV4 plays a role in mediating the stretch induced stress on human lung epithelial cells and macrophages, we wanted to examine the effect of a TRPV4 antagonist in a murine mechanical ventilation *in vivo* model with high tidal volumes. No increase in cytokine release or protein concentration in BALF was observed when mice were subjected to a tidal volume of 20 ml/kg. However, a tidal volume of 30 ml/kg ventilation resulted in a 2.6 fold increase protein concentration in BALF compared to the normal ventilated control group (Fig 6A). IL-6 release was 15.3 fold higher (Fig 6C) after mechanical ventilation (30 ml/kg T_V_) and we observed a 23.3 fold increase of KC/GRO level (Fig 6B) compared to control group (6.5ml/kg TV). All were significantly blocked with the TRPV4 inhibitor.

## Discussion

Despite its lifesaving potential, mechanical ventilation may induce lung damage by itself. Lung strain during mechanical ventilation is poorly defined and difficult to estimate because of the heterologous local lung susceptibility. Therefore lung areas may receive higher tidal volume than estimated leading to massive overdistension of lung cells. In turn areas that receive the higher tidal volume, may promote a local inflammatory response assumed to have the potential to induce VILI [6, 7]. In this study we investigated the potential of a TRPV4 inhibitor for the improvement of mechanical ventilation induced pathological response in lung cells, using the TRPV4 antagonist GSK2193874 in both *in vitro* and *in vivo* models of pathophysiological stretch.

Human lung epithelial cells (NCI-H292) showed a significant TRPV4 agonism evoked Ca^2+^ response that could be concentration-dependently reduced and blocked by TRPV4 antagonism. NCI-H292 also showed an extension-evoked Ca^2+^ response that could be significantly reduced by 26% with TRPV4 inhibition. This is consistent with previously reported data in which TRPV4 mediated stretch-evoked Ca^2+^ influx contributes to the increase membrane permeability due to lung over-distention following high PIP ventilation [36] and in mice primary urothelial cell, where the Ca^2+^ increase was partially reduced in TRPV4KO-type compared to WT cells during stretch stimuli [30]. The increase in Ca^2+^ concentration could not be completely blocked by TRPV4 antagonism and assume that the TRPV4 independent calcium influx could be regulated by other mechanosensory channels and systems, perhaps with different thresholds, that might play a role on the initial calcium response of the lung epithelium to extension.

In these experiments, cells had to be stretched with a magnitude of 80% within 400 ms in order to induce TRPV4 activation. One possible reason for the need of such large stretch amplitude is that in these experiments the mechanical strain system stretches cells in a uni-axial direction, compared to the multidirectional extension in the mechanically ventilated lung *in vivo*. Another explanation may be that these cellular experiments were performed at room temperature (due to technical reasons), whereas in the lung, the epithelial temperature, particularly in the lower airways, is likely to be higher, which may impact the heat-sensitive TRPV4 channel. The need for large stretch amplitudes for the TRPV4 mediated strain-evoked calcium entry was also observed on mice primary urothelial cells under similar conditions [30].

VILI has been described as a cellular response to mechanical stress that includes a rapid increase in vascular permeability followed by cytokine release [3, 37]. Deformation per se can trigger inflammatory signalling and it is possible that alveolar epithelial cells may play an active role in ventilator-induced lung injury [15]. We further tested the hypothesis that the initial calcium response to stretch results also in an inflammatory response in human epithelial cells. TRPV4 activation of epithelial cells with the synthetic TRPV4 agonist GSK1016790A resulted in a release of the pro-inflammatory cytokines IL-6 and IL-8 *in vitro* that could also be completely blocked by addition of the TRPV4 antagonist GSK2193874. This is consistent with a recent study in fetal mouse distal lung epithelial cells which demonstrated that TRPV4 may play an important role in the transduction of mechanical signals in the lung epithelium by modulating the release of the cytokine IL-6 via p38 and ERK pathways [31]. Cyclical stretching of epithelial cells in equibiaxial direction also increased release of the cytokines IL-6 and IL-8 after stretch that could also be reduced by about 30% with GSK2193874 and was decreased by about 80% by addition of the general TRP-channel blocker Ruthenium Red, suggesting that this effect is only partially modulated by TRPV4 and other stretch activated channels and integrins may play a role. It is not unreasonable that the applied stretch of 30% in our studies mimic the deformation by the lung epithelium in situ when mechanically ventilated with recommended ventilator settings [38] during which the lung volume more than doubles. Interestingly the stretch-evoked increase in intracellular calcium on NCI-H292 that was reduced by 26% with TRPV4-antagonist addition is similar to the about 30% reduction of the stretch induced cytokine release via TRPV4 antagonism, suggesting that the size of changes in Ca^2+^ influx extrapolate directly to cytokine release.

Macrophages are a major source of cytokine secretion and are known to adhere directly to lung epithelial cells [39] and because of this property may also be exposed to stretch during mechanical ventilation, and have indeed been showed to be activated by strain *in vitro* resulting in an increase in IL-8 [14]. Therefore we examined the effect of mechanical stretch on isolated human macrophages *in vitro*. The mechanical stretch induced stress on the pro-inflammatory M1, and to a lesser extent in the tissue remodelling M2, macrophages resulted in an significant increase of the cytokines IL-1α, IL-1β and the chemokine MCP-1 and also small but significant increases in IL-6 and -8. Interestingly, and in contrast to the findings in epithelial cells, the stretch induced increase of the cyto- and chemokines levels could be nearly completely abolished by the TRPV4 antagonist. Particularly interesting is that IL-1α has been showed to directly increase vascular endothelial cell permeability *in* vitro [40], and therefore may play an important role in the stretch induced pulmonary vascular permeability increase reported during ventilation leading to VILI. We have recently shown that macrophage-derived IL-1α and IL-1β promotes permeability increases in primary human epithelial cells differentiated in air-liquid interface [41], indicating that the TRPV4 mediated stretch induced release of cytokines from macrophages could not only affect the vascular endothelium but also directly act on the lung epithelium permeability increase leading to edema formation and alveolar flooding during ventilation that may induce or aggravate VILI. M1 macrophages may mimic an alveolar phenotype because they express PPARγ that has been shown to be an alveolar macrophage marker [42]. Furthermore, in the hyperinflammatory environment associated with ARDS, macrophages are more likely to be driven initially towards a pro-inflammatory, rather than a tissue remodelling phenotype and TRPV4 has also been reported to mediate polarization of macrophages toward an M1-like phenotype [21, 22, 43].

However, the signal transduction pathway from a mechanical stimulus resulting in an inflammatory response remains elusive. One possibility is that TRPV4 inhibitors blocks other Ca^2+^ dependent processes, such as the release of cytokines. TRPV4 has also been showed to play an role in the transduction of mechanical signals in the distal epithelium by modulating inflammation (IL-6 secretion) via p38 and ERK pathways [31]. Release of inflammatory cytokines IL-6 and -8 after TRPV1 activation has been showed to be mediated through MAPK signalling in corneal epithelium [44]. These two pathways may also be important downstream activators of TRPV4 in mediating an inflammatory response after stretch.

Having demonstrated that both lung epithelial cells and macrophages play an active role in the TRPV4-mediated stretch induced cytokine release, we wanted to evaluate the effect of the TRPV4 antagonist GSK2193874 in a murine model of mechanical ventilation similar to other model promote to induce lung injury [45, 46]. The high tidal volume ventilation (30 ml/kg T_V_) protocol significantly enhanced protein, IL-6 and KC/GRO concentrations in BALF compared to the normal ventilated group (6.5ml/kg T_V_), all of which could be nearly completely blocked with the TRPV4 antagonist. These data are consistent with the *in vitro* findings in macrophages, which were almost entirely TRPV4-dependent, but less so with the *in vitro* epithelial findings, which were only partially dependent on TRPV4. This may suggest that macrophages are stronger effector cells during ventilation contributing to a pathological response compared to epithelial cells, a hypothesis which is consistent with the findings of others [19, 47, 48] and that these effects are TRPV4-dependent. An important role for alveolar macrophages in mechanical ventilation models has also been demonstrated by depletion of macrophages in rat lungs using clodronate-filled liposomes resulting in an attenuation of ventilator-induced lung injury, where high volume ventilation resulted not only in a activation-associated adhesion of alveolar macrophages but also in an increased alveolar protein leak and lung edema formation that was attenuate by depletion of macrophages [47, 48]. A more recent investigation linked TRPV4 channels and macrophages in the role of modulating VILI. In this study the ventilator induced lung injury was markedly attenuated in TRPV4KO mice, whereas reintroduction of TRPV4WT macrophages in TRPV4KO mice reconstituted the lung injury response to mechanical ventilation showing that macrophages TRPV4 activation plays a crucial role in initiating this injury [19]. One study demonstrated that the inflammatory response to particles was amplified by contact-dependent interactions between alveolar macrophages and epithelial cells [39] and it is also possible that a similar interaction occurs during ventilation. The importance of TRPV4 in preventing VILI was indirectly addressed in another study in which inhalation of nanoparticles containing Ruthenium Red prevents ventilator damage and vascular permeability for several days [49]. However Ruthenium Red is known to impact calcium handling in cells via effects on other TRP channels [50–52] and non-TRP proteins [53–56] and it therefore remains a possibility that the effect of Ruthenium Red nanoparticles on ventilator-induced oedema in this study may be modulated via other TRP channels or via modulation of downstream intracellular calcium handling [49].

In summary we showed that mechanical stretch evoked intracellular Ca^2+^ influx and induces the release of pro-inflammatory cytokines that was partially dependent upon TRPV4 in epithelial cells, but also induced the release of pro-inflammatory cytokines from M1 that was entirely dependent upon TRPV4. In a murine ventilation model with high tidal volumes, TRPV4 inhibition attenuated pulmonary barrier permeability increase and pro-inflammatory cytokines secretion. Taken together, these data suggest TRPV4 inhibitors may have utility as a prophylactic pharmacological treatment to improve pathological responses of lung cells exposed to stretch during ventilation and potentially may have utility in the support of patients receiving mechanical ventilation.
